# Characterisation of Drug Delivery Efficacy Using Microstructure-Assisted Application of a Range of APIs

**DOI:** 10.3390/pharmaceutics12121213

**Published:** 2020-12-15

**Authors:** Raha Rahbari, Ionut Ichim, Ryan Bamsey, Jemma Burridge, Owen J. Guy, John Bolodeoku, Michael Graz

**Affiliations:** 1 Singleton Campus, Institute of Life Science 2, Swansea University, Innoture Ltd., Swansea SA2 8PP, UK; Iichim@innoture.co (I.I.); RBamsey@innoture.co (R.B.); jburridge@innoture.co (J.B.); jbolodeoku@innoture.co (J.B.); m.graz@innoture.co (M.G.); 2 Chemistry Department, Swansea University, Swansea SA2 8PP, UK; o.j.guy@swansea.ac.uk

**Keywords:** transdermal drug delivery, microneedles, microstructures, skin penetration, drug permeation enhancement

## Abstract

Polymer-based solid microstructures (MSts) have the potential to significantly increase the quantity and range of drugs that can be administered across the skin. MSt arrays are used to demonstrate their capacity to bypass the skin barrier and enhance permeability by creating microchannels through the *stratum corneum*, in a minimally invasive manner. This study is designed to demonstrate the ability of MSts to exceed the current boundaries for transdermal delivery of compounds with different molecular weights, partition coefficients, acid dissociation constants, melting points, and water solubilities. In vitro permeation of a range of selected molecules, including acetyl salicylic acid (aspirin), galantamine, selegiline hydrochloride (Sel-HCl), insulin, caffeine, hydrocortisone (HC), hydrocortisone 21-hemisuccinate sodium salt (HC-HS) and bovine serum albumin (BSA) has been studied across excised porcine skin with and without poke and patch application of MSts. Permeation of the molecules was monitored using Franz diffusion cells over 24 h. MSts significantly increased the permeation of all selected molecules up to 40 times, compared to topical applications of the molecules without MSts. The greatest increase in permeation was observed for caffeine with 70 ± 8% permeation and the lowest enhancement was observed for HC with a 2.4 ± 1.3% increase in permeation. The highest obtained flux was BSA (8133 ± 1365 μg/cm^2^/h) and the lowest flux observed for HC (11 ± 4 μg/cm^2^/h). BSA and HC also showed the highest (16,275 ± 3078 μg) and the lowest (73 ± 47 μg) permeation amount after 24 h respectively. MSt-treated skin exhibits greatly increased permeation. The molecule parameters (size, acid dissociation constant, partition coefficient and solubility)—traditional hurdles associated with passive diffusion through intact skin—are overcome using MSt skin treatment.

## 1. Introduction

Transdermal drug delivery systems are a highly promising method for drug administration, and an alternative drug delivery method to oral administrations. Transdermal drug delivery offers many benefits over oral administration including: circumventing the first pass metabolism through the gastrointestinal tract; reducing cytotoxicity and side effects of drugs; evading enzymatic reactions of drugs in the stomach and liver; reducing drug fluctuation levels in plasma; and increasing the absorption and bioavailability of drugs [1,2,3].

Although there are important clinical advantages for topically applied formulations, drug administration via the skin is still quite infrequently used as a first-line approach. This is due to the outermost layer of the skin, the *stratum corneum*, being a highly effective barrier to molecules and drugs, preventing entry of substances into the skin and their subsequent entry into systemic circulation [4,5].

Circumventing this skin barrier introduces a highly beneficial delivery route for drug delivery into the body.

The permeation of drugs from the surface of the skin into systemic circulation involves steps of entry and partition of pharmaceuticals into the different skin layers, depending on the skin permeation routes (transcellular route; the intercellular pathway and permeation via appendages [6,7]). The entire diffusion and partition process is complex, and can be affected by multiple factors including: skin physiological factors, such as age, body site and race [8]; or physicochemical properties of drug formulations, such as partition coefficient, molecular size, solubility, melting point and ionisations [9,10,11]. For these reasons, so far, only 20 drugs have shown significant skin permeation suitability [12]. These molecules predominantly have properties such as low molecular weight, intermediate hydrophilicity/hydrophobicity, and partition coefficients between 1 and 3, which are appropriate for skin permeation. Thus, transdermal delivery of drugs, with one or more parameters outside these confines, through intact skin presents significant challenges in providing sufficient therapeutic doses, particularly when considering drugs with low potency [13,14].

Transdermal delivery through intact skin using patches or chemical enhancers can also be a slow process, limited by diffusion rates. A fast delivery process may be more desirable in certain applications such as pain relief. An example of the complexity of transdermal drug delivery is the binding of drug molecules to the keratin content within the *stratum corneum* and subsequent formation of a drug reservoir within the skin membrane. This concept is well established, especially for steroidal drugs such as hydrocortisone, which tend to remain in the stratum corneum layer [15]. In addition, as the skin is metabolically active, there is also potential for drugs to be degraded by enzymes or hydrolysed within the skin layers, thus preventing systemic entry. Furthermore, depending on the physiochemical structure of the drugs, they may be maintained within the skin layers or enter into the subcutaneous fat layer, rather than entering into blood circulation [15].

To overcome transdermal delivery challenges and bypass the *stratum corneum* there are several possible drug administration approaches. Injections are an efficient way of bypassing the *stratum corneum*, but cross contamination, needle phobia, painful and repetitive injections and collapsing veins are just a few reasons for the unpopularity of needle injections [16,17,18]. Microneedles (MN) are an alternative and very effective way to avoid these problems.

MNs are non-invasive and relatively painless when applied. They can be self-administered and increase patient compliance. MNs create microchannels in the skin, thus bypassing the stratum corneum and dramatically increasing the number of compounds that can be administered across the skin, including bio-therapeutic vaccines, drugs and other molecules (especially large hydrophilic molecules [16,19], which have great difficulty in passing through intact skin). There are several types of MNs that can be used for transdermal delivery systems, including solid, hollow, coated, dissolvable and hydrogel MNs [18,20,21,22]. MNs can be made from polymer, metal, silicon, glass or ceramics, with different insertion sizes and shapes possible [23]. Solid MNs followed by application of drug formulation in a “poke and patch” approach is one of the most straightforward and widely studied MN-drug delivery methods [24,25]. Depending on the type of MN and materials used, the MN manufacturing process can be quite challenging and time consuming. For instance, bulk micromachining of dissolvable MNs with appropriate needle architecture is a challenging process. Reactive ion etching to develop silicon wafers containing MNs can also be a costly processing method, which can take hours or days to complete [26]. Other challenges include a limited choice of appropriate biomaterial, insufficient mechanical strength and multi-step production techniques and poor control over drug delivery [27]. For this reason, many MN production techniques remain as research topics and never reach commercialisation.

Solid polymer-based MN arrays, described hereafter as microstructures (MSts), since the manufacturing technique, material and resulting structure are non-derivative and distinctly different to any other MN based devices, are used for transdermal drug delivery development via disruption of the *stratum corneum*. MSts have been manufactured via microlithographic 3D printing (ML3DP) using stainless steel stencils and a range of biocompatible materials. The novel ML3DP method uses sequential deposition and UV curing steps to build up the MSt structure over a large or small area substrate. These unique MSt patches are highly scalable and extremely cost effective for immediate mass production. Furthermore, MSts are fully customisable and can be formed on a range of different base substrates with any shape and size and thus, can conform to different anatomical body sites. The patented MSt devices are based on the same manufacture platform used for production of Radara^®^, a commercially available facial cosmetic MSt product, used for delivery of hyaluronic acid (HA) for skin rejuvenation and wrinkle reduction. Clinical studies using Radara^®^ (ENHANCED-1 and ENHANCED-2) have previously confirmed efficacy of this product in terms of reduction in facial skin wrinkles and increased moisture and elasticity level.

This study presents an investigation of the efficacy of MSts for enhancing transdermal delivery of a range of drug molecules, including acetyl salicylic acid (aspirin), galantamine, R-(-)-deprenyl hydrochloride (also known as selegiline hydrochloride) (Sel-HCl), insulin, caffeine, hydrocortisone (HC), hydrocortisone 21-hemisuccinate sodium salt (HC-HS) and bovine serum albumin (BSA). The molecules selected have a range of different physicochemical properties in terms of molecular size, such as partition coefficient (LogP), acid dissociation constant (pKa), melting point and solubility in water (Table 1).

Within these physicochemical properties’ limits for existing drugs, the selected molecules span across the range of values. The molecules chosen would also benefit from being delivered transdermally.

Insulin was selected as a candidate molecule because it is a hormone with a relatively large molecular weight (5808 Da), and there are significant potential benefits from delivering insulin in a minimally invasive transdermal application. Most studies on transdermal delivery of insulin have been performed in vivo, since insulin has high solubility in plasma [28,29,30,31].

Caffeine with logP −0.07 and pKa 10.4 was selected as a hydrophilic molecule, which would benefit from MSt assisted delivery, as caffeine has been widely studied with successful skin permeability using MNs [32,33].

Aspirin is commonly delivered orally, with widely known side effects. There are clear benefits to delivering aspirin transdermally, since this would bypass the gastrointestinal tract, but there are few reported studies relating to MN-assisted delivery of aspirin [34,35]. Thus, aspirin as a weak acid was selected to assess the capability of a molecule with low pKa (3.5) for transdermal delivery using MSts.

The lipophilic outermost layer of the skin only allows moderately lipophilic, potent, and small molecules to passively permeate into deeper layers. This highly limits transdermal delivery of large molecular weight proteins which have a complex properties with acid-base side chain and are highly polar in nature [36,37]. Thus, delivering proteins transdermally is highly challenging. Although BSA (with molecular weight of 66,000 Da and 300 mg/mL water solubility) is not a drug molecule, it has been commonly used as a model to study large, hydrophilic protein permeation across the skin [38,39]. Thus, BSA was selected to assess permeation efficacy of large molecular weight proteins in conjunction with MSts.

Galantamine was selected as it is a weak base with pKa 8.2 and intermediate partition coefficient (logP 1.8), and is one of only five FDA approved drugs for treatment of Alzheimer’s [40]. There are many side effects related to oral consumption of galantamine (vomiting, anorexia, nausea and abdominal pain). This, combined with galantamine’s molecular properties, high bioavailability and low daily dose requirement, make this drug an attractive candidate for transdermal drug delivery [40]. The effect of formulation factors such as enhancers and pressure sensitive adhesives for transdermal delivery of galantamine have been previously investigated [41,42,43]. However, transdermal delivery of galantamine using MSts is a novel approach.

Sel-HCl, as one of only twenty approved transdermal drugs on the market [44,45] (for treatment of major depressive disorders, as well as Parkinson’s disease [46,47]), was selected due to its intermediate physicochemical properties and its side effects (acute hypertensive reactions following ingestion of food containing high tyramine concentrations [48]) when orally ingested. The combination of Sel-HCl with MSts for transdermal delivery is a novel approach.

HC (the steroid) was selected due to its wide multifunctional therapeutic application (including acute inflammation, asthma and skin conditions [49,50]). Whilst transdermal delivery of HC using cream, adhesive patches [51], micro/nano-encapsulations [52,53] and phonophoresis [54] have been previously studied, application of HC using MSts is a new approach (although other steroids have been reported to be delivered via MN [55]), which could greatly enhance the permeability of this drug. In this study, the in vitro permeation of both HC (pKa 13.8) and HC-HS (pKa 5.64) have been performed to compare the efficiency of using MSt in delivery of both formulations.

This study is designed to characterise the degree of enhancement of transdermal drug delivery for a range of APIs with varied physicochemical parameters. Several of the molecules selected demonstrate the challenges encountered in transdermal diffusion across the intact skin barrier. The enhanced transdermal diffusion using MSts is characterised and compared to diffusion of molecules through intact skin.

Note that only figures for solubility have been directly measured in this work and that all other values reported in Table 1 are extracted from the literature (Pubchem). Variability in reported values is related to whether they are experimentally determined or theoretically derived, and whether they are synthesised by different manufacturers.

## 2. Materials and Methods

### 2.1. Materials

Chemicals including acetic acid (45754-500ML-F), trifluoroacetic acid (TFA) (302031-100ML), HPLC grade methanol (34860-2.5L-R) and acetonitrile (34851-2.5L) and water (270733-2.5L), acetyl salicylic acid (A5376-100G), hydrocortisone (H4001-1G), hydrocortisone-21-hemisuccinate sodium salt (H4881-100mg), insulin human recombinant (91077C-100mg), R-(-)-deprenyl hydrochloride (M003-250mg) and fluorescein hyaluronic acid (F1177-5mg) were purchased from Sigma Aldrich UK. BSA (P06-1391100) was supplied by Pan Biotech. Galantamine was kindly provided by Swansea University, College of Medicine. 1 mM Vybrant™ Dil cell-labelling solution was obtained from Thermo Fisher Scientific (V22885, Waltham, MA, USA). Detergent Compatible (DC^TM^) protein assay (5000112) was purchased from Bio-Rad. Medical grade urethane acrylate polymer, was obtained from Intertronics (Oxfordshire, UK). Polyvinyl chloride (PVC) film was obtained from Amazon. Excised porcine skin from a large white piglet (approximately eight weeks old) was obtained from WetLab-Medmeat (Warwickshire, UK), prior to cleaning, compliant with the ABPR REG EEC 142/2011. Excised human abdominal skin (female donors) was obtained from Ethical Tissue, the Research Tissue Bank (University of Bradford, Bradford, UK) under full ethical approval from the Leeds (East) Research Ethics Committee (REC ref. 17/YH/0086).

#### 2.1.1. Microstructure Patch Production Method

An array of MSts were produced via ML3DP using a screen printer (Adopt SMT, Grödig, Austria). Custom made electroformed or laser cut stencils were obtained from ASM (Weymouth, UK). The polyurethane foam or PVC base substrate was placed onto a shuttle which was positioned on the printer using fiducial marks, allowing it to be repositioned every printing pass.

**Figure 1:**
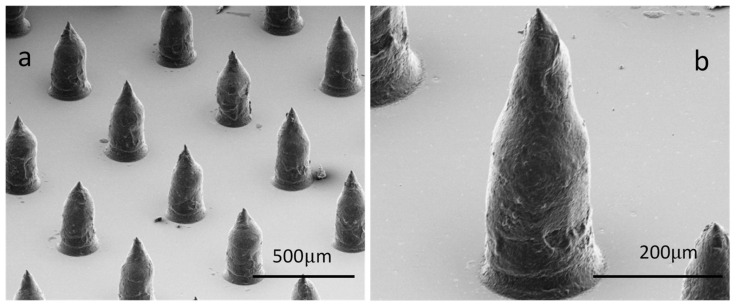
(**a**,**b**) show SEM images of different magnifications of microlithographic 3D printed microstructures on polyvinyl chloride (PVC) base substrate.

Urethane acrylate polymer gel was pushed across the surface of the stencil using a squeegee blade, forcing the polymer through the stencil “apertures”, and depositing a small amount on to the base substrate below the stencil (see Figure 1). Once the polymer had been deposited, it was cured under UV light (365–405 nm) for 1–2 s. This process was repeated multiple times to build up the MSts.

#### 2.1.2. Skin Sample Preparation

Porcine skin samples were used in this study, since it is the closest model to human skin, and is suitable for trimming using an electric dermatome, thus producing less variability in skin thickness. Pig skin was purchased from WetLab-Medmeat supplies, where animals are slaughtered in accordance with all relevant regulations (ABPR REG EEC 142/2011). The skin samples, from a whole suckling with non-scalded skin, have an undamaged *stratum corneum* as the animal is not exposed to hot water. Freshly slaughtered animals were delivered frozen. The whole animal was then defrosted, its hair shaved using hair clippers and the skin trimmed to approximately 500 μm thickness using a dermatome (Integra Life Sciences™, Padgett Instruments, Plainsboro, NJ, USA). The skin samples were then stored at −20 °C freezer until the time of experiment. No skin samples with wounds, warts or hematomas were utilised. Samples were used within two months of slaughter.

Human skin was used as supplied, with full thickness, for histological analysis of MSt penetration and subsequent drug models (fluorescent dyes) diffusion.

#### 2.1.3. Skin Treatment Using MSts

Excised skin samples (approximately 3 × 3 cm) were placed on a cork board covered with tin foil. Skin samples were then treated by manual poke and patch application of MSts, using an applied force of 40 N for 60 s, in all skin testing studies (including characterisation and permeation studies). For in vitro permeation studies (for all samples and controls) the skin samples were sandwiched in Franz cells, prior to administration of the drug samples onto the surface of the skin samples.

### 2.2. Characterisation of MSts and Skin Penetration

#### 2.2.1. MSts Penetration Efficacy

The penetration efficacy of MSts into skin layers was assessed for both porcine and human skin. 4 cm^2^ PVC base substrate MSt patches were manually applied to the full thickness (skin that had not been dermatomed, with approximately 1000 μm thickness) excised skin samples. The treated skin samples were then transferred immediately into a freezer and maintained at −20 °C for 24 h to ensure the samples were frozen. Skin samples were then prepared for microsectioning (30 μm) using a cryostat.

#### 2.2.2. Monitoring the Diffusion of Fluorescent DiI Using MSts

Vybrant™ Dil solution was encapsulated within phospholipid-based vesicles, using a modified version of the “thin film hydration sonication” technique, described by Opatha et al. [56]. Encapsulation of DiI (5 μL of 1 mM), using l-α-Phosphatydilcholine and Tween-80 surfactant (1% *w*/*w*) dissolved in chloroform/methanol (2:1) solvent mixture, was performed in order to resolve the solubility issue of the water insoluble DiI dye, to facilitate skin permeation using MSt and improve confocal imaging.

The suspension was centrifuged to separate the encapsulated DiI from the un-encapsulated DiI, with the supernatant resuspended in PBS buffer and then sonicated in order to produce the desired vesicle size (ranges of 80–100 nm). The resulting encapsulated DiI (E-DiI) was then used in combination with MSts in a poke and patch procedure to monitor the diffusion of E-DiI into the deeper skin layers.

Excised human skin was cut into 4 cm^2^ sections and placed on wet cotton cellulose tissues to avoid dehydration of the excised skin samples. Skin samples were treated with MSt-assisted E-DiI in a poke and patch procedure. 4 cm^2^ PVC base substrate MSt patches were used to treat the skin samples. Subsequently, skin samples were treated with 100 μL of E-Dil, following removal of the MSt patches from each skin sample. The skin samples remained on wet cellulose tissues at room temperature for 3 h, in order for E-Dil to diffuse within the deeper layers of skin via the microchannels created by the MSts. The treated skin samples were then transferred to a −20 °C freezers, in order to prepare for microsectioning using a cryostat. A Zeiss LSM 710 confocal microscope (Carl Zeiss, Oberkochen, Germany) was used to examine the skin sections at an emission wavelength of 543 nm.

#### 2.2.3. Monitoring the Diffusion of Fluorescein Hyaluronic Acid Using MSts

Skin permeation of fluorescein hyaluronic acid (F-HA) (molecular weight of 800,000 Da) using MSt. 100 μL of 1 mg/mL F-HA was applied on excised human skin, following the application of MSt visualised using confocal microscopy at 488 nm. F-HA was maintained on the surface of the skin sample for 3 h to ensure the diffusion of F-HA into the skin layers.

#### 2.2.4. In Vitro Permeation Studies of APIs

In vitro permeation studies were performed using vertical Franz diffusion cells (Soham Scientific, Ely, UK) with an effective diffusion area of 1.77 cm^2^ and nominal cell volume of 4 mL. The receptor chambers were filled with degassed PBS (pH 7.4) and the magnetic stirrer was set at 400 rpm to maintain the receptor solution homogeneity. The temperature of the Franz cells was set and maintained at 32 °C. Porcine skin samples were placed between the donor and receptor chambers of the cell with the dermal side in contact with the receptor medium. Samples were collected at time 5, 15, 30, 60, 120, 240 and 1440 min and the equivalent sample volume replaced with fresh PBS solution. Nine different permeation repeat studies per drug solution were performed using porcine skin. The concentration of each of the permeated drugs (aspirin, galantamine, HC, HC-HS, sel-HCl, insulin and caffeine) in the receptor compartment of the Franz cell was analysed at each time point using HPLC. The concentration of permeated BSA protein was analysed using protein quantification assay. The cumulative permeated amount for each drug sample was plotted against time.

#### 2.2.5. Drug Solubility and Choice of Franz Cell Receptor Buffer

The aqueous solubility of drugs is critical in terms of in vitro permeation experiments, since drugs need to be delivered across skin into the receiver compartment of Franz cells, containing aqueous buffer (e.g., PBS). Adequate solubility of the drugs in the receptor fluids was tested to ensure it was not a barrier to permeation. The use of physiologically conductive Franz cell receptor fluid (PBS) was preferred for the majority of drugs, except for insulin, which is insoluble in PBS and water with pH 7.4 (Table 2). For insulin, an alternative solvent, 0.01 M HCL, was thus used to assess solubility and subsequently used as the receptor fluid for this drug. HC is also insoluble in water and thus, ethanol was used to solubilise this drug. However, the dissolved HC in ethanol was solubilised in receptor fluid containing PBS for HPLC studies. All drugs were solubilised at room temperature, except aspirin, which was dissolved in PBS and water at 40 °C. The initial amount of each drug used was dictated by each molecule’s solubility and ease of detection.

### 2.3. Detection and Quantification of Molecules

#### 2.3.1. Detection and Quantification of BSA Using a Colorimetric Assay

Detection of BSA in the receiver compartment of the Franz Cell was determined using a DC^TM^ protein colorimetric assay and quantified by reference to the serial dilution standard UV/Vis absorption curve of BSA, produced using an UV/Vis spectrophotometer plate reader (FLUOstar Optimal, Richmond Scientific, Chorley, UK). 5 µL of Franz cell solution was used to quantify the amount of permeated BSA. The detection and quantification procedure was followed based on the protocol provided by the supplier [57].

#### 2.3.2. Detection and Quantification of APIs Using HPLC

A simple, rapid and specific method using HPLC with ultraviolet (UV) detection was developed for each of the drugs used in this study (Table 3). The methods were validated for each drug sample based on linearity, precision, and accuracy of their standard curves. The lower limit of quantification (LLOQ) over the concentration range of 1–0.001 mg/mL for each drug sample was determined. The coefficients of determination (R^2^) were expressed as ±SD (*n* = 3). The desired standard required R^2^ greater than 0.99 and the standard deviation to be within ±15% for all the concentrations tested. A C18-column (150 mm, 4.6 mm, particle size 5 μm; Phenomenex, UK), equipped with a guard column of the same packing and was used to analyse all molecules (aspirin, galantamine, HC, HC-HS, sel-HCl, insulin and caffeine). All analyses were performed in isocratic mode with a flow rate of 1 mL/min, injection volume of 10 μL, at room temperature.

#### 2.3.3. Calibration Curve

Quantification of each drug was derived from the specific peak area referenced to the concentration calibration curves for each drug respectively. Three different calibration standards were prepared in total for each calibration curve (with R^2^ of above 0.99). The calibration range of 1–0.001 mg/mL for each drug sample was chosen to correspond to the concentration of each drug in the receptor medium after in vitro drug delivery.

#### 2.3.4. Data Analysis

The individual permeation profile of each drug molecule was obtained by plotting the cumulative amount of the molecule (μg) in the receptor fluid against time (h). The maximum flux value (J) at steady state (μg/cm^2^/h) represents the slope of the linear portion of the cumulative plot, (which is determined by the linear segment with the highest linearity coefficient value (R^2^), divided by the surface area (1.77 cm^2^) [42,58]. The enhancement ration (ER) was calculated according to the equation:

ER = % permeation of molecules with MSt/% permeation of molecules without MSt

All data were expressed as ± standard error of nine replicate. A one-way ANOVA analysis to study the statistical significance between formulations was conducted with the software Prism (version 6).

## 3. Results

### 3.1. Characterisation of MSts and Skin Penetration

#### 3.1.1. Characterisation of MSt Dimensions

The manufacturing method of MSt (ML3 DP) is a high throughput production technique, which enables multiple MSt patches to be produced simultaneously on a large volume scale. Figure 1a,b show different magnifications of the MSts which are uniform and have sharp apex of approximately 17 ± 2 µm. The height of MSts are approximately 500 µm with the pitch, dictated by the MSts stencil spacing of 800 µm.

#### 3.1.2. MSt Penetration Efficacy

**Figure 2:**
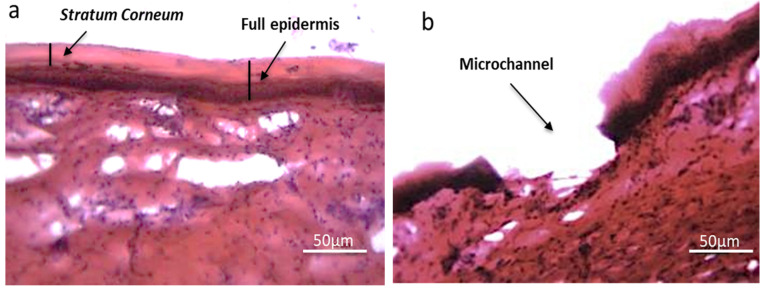
Optical microscopy images of porcine skin treated with microstructure (**b**), compared to untreated porcine skin (**a**).

Skin sections histologically visualised using optical microscopy (Figure 2) provide qualitative skin permeation data. Figure 2b shows disruption of the stratum corneum and successful penetration of MSt into viable epidermis, following application and removal of the MSt, compared to untreated porcine skin (Figure 2a).

#### 3.1.3. Characterisation of DiI Skin Permeation Using Polymer-Based Solid Microstructures (MSts).

LSCM was used to qualitatively investigate the penetration efficacy of MSt into excised human skin and diffusion of the drug model (E-DiI) into deeper skin layers.

**Figure 3:**
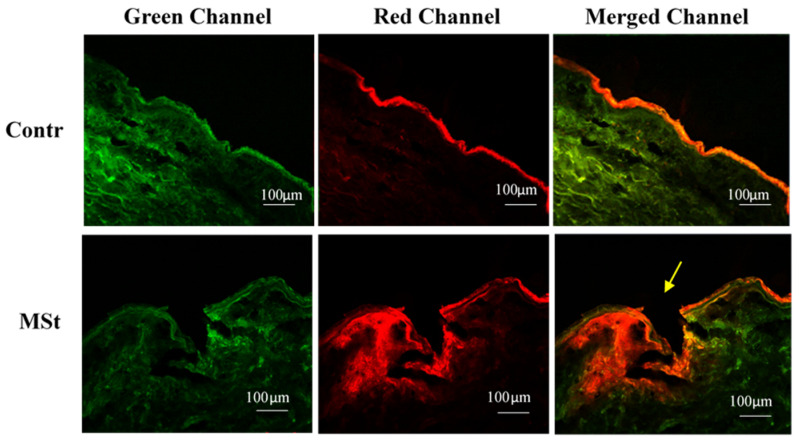
LSCM images of human skin treated with E-DiI following application of microstructures, compare to control (treatment without microstructures). Green intensity is due to auto-fluorescence of the skin sample. Red intensity indicates diffusion of E-DiI around the microchannel area created by MSt insertion. Microstructures enhanced the diffusion of E-DiI into deeper skin layers.

DiI is water-insoluble and it does not diffuse into higher water content skin layers such as the viable epidermis and dermis. To counteract this issue, phospholipid vesicles were used to resolve the solubility of DiI. Encapsulation of hydrophobic dye in order to study MN characteristics and permeation enhancement has been previously reported (encapsulation improves the solubility of poorly water-soluble molecules) [59,60]. Delivery of fluorescent drug model (E-DiI) via microchannels created by MSt application was investigated using LSCM of TF-DiI applied on the skin sample following the application of MSts. Figure 3 shows the diffusion of the TF-DiI in the skin region around the micro channel formed within the viable epidermis by MSt application, in comparison to a control sample where TF-DiI was applied without MSt treatment and remains in the SC layer.

#### 3.1.4. Characterisation of F-HA Skin Permeation Using MSts

**Figure 4:**
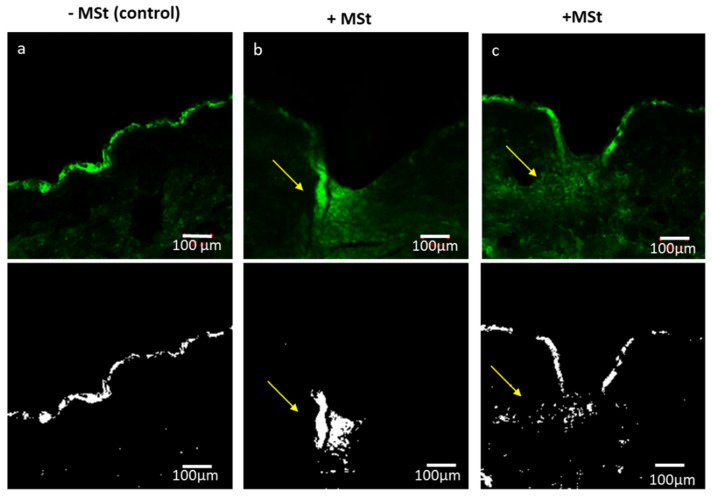
LSCM images of human skin treated with F-HA histological analyses show: (**a**) control sample where FITC-HA was applied without microstructure treatment and remained at the surface of the skin, in comparison to the diffusion of the FITC-HA (MW = 800 kDa) into the deeper layers of excised human skin samples, in the vicinity of the microchannels created by microstructure application (**b**,**c**). (Yellow arrows indicate the diffusion of HA into deeper skin layers using MSts. Skin is auto-fluorescence (green intensity); however, the intensity of FITC-HA is higher compared to the skin background. The black and white images have background fluorescence removed and show diffusion of HA into the deeper layers of skin samples using microstructures. Scale bar = 100µm.

To evaluate the feasibility of the transdermal delivery of large molecular weight drugs, F-HA (with molecular weight of 800,000 Da, LogP of −2.6 and pKa of 4) was applied on the surface of excised human skin following the application of MSt. LSCM enabled visualisation of the distribution of the F-HA into the deeper skin layers following MSt application (Figure 4b,c), as opposed to application of F-HA without MSt (Figure 4a). Figure 4a shows F-HA remained on the surface of the skin and no diffusion was observed without MSt application.

### 3.2. In Vitro Permeation Studies

**Figure 5:**
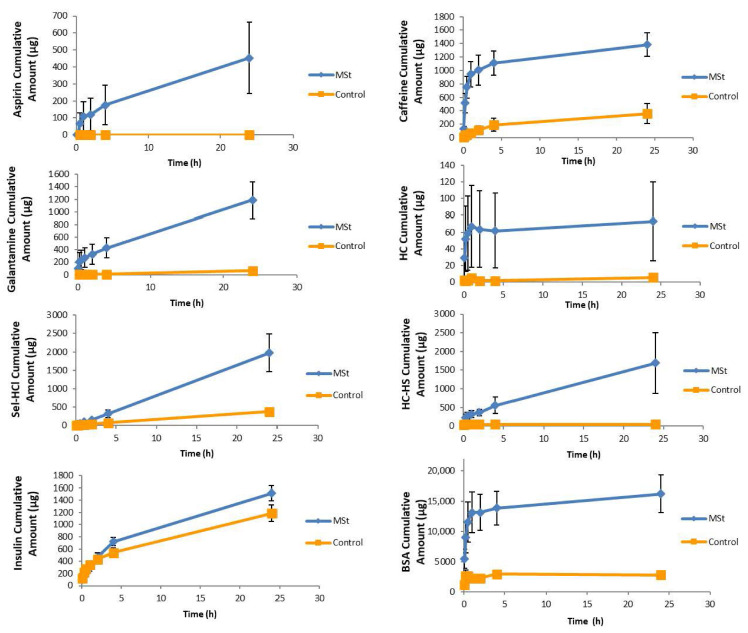
Permeation profile for eight investigated molecules (BSA, aspirin, galantamine, caffeine, HC-HS, HC, insulin, selegiline) using porcine skin and Franz diffusion cells. The skin temperature was maintained at 32 °C ± 1 during the 24 h. Each point represents the mean cumulative amount in μg ± standard error expressed for the investigated dosage form (*n* = 9).

Nine replicates were performed for each molecule (BSA, aspirin, galantamine, caffeine, HC-HS, HC, insulin and sel-HCl) under the same (except for the solvent issues) experimental conditions. Table 4 shows the cumulative permeated amount, flux, permeation percentage and enhancement rate of each molecule. Figure 5 shows the cumulative amount of drug, expressed as ±SE (standard error), that permeated through porcine skin over 24 h with and without using MSts.

**Figure 6:**
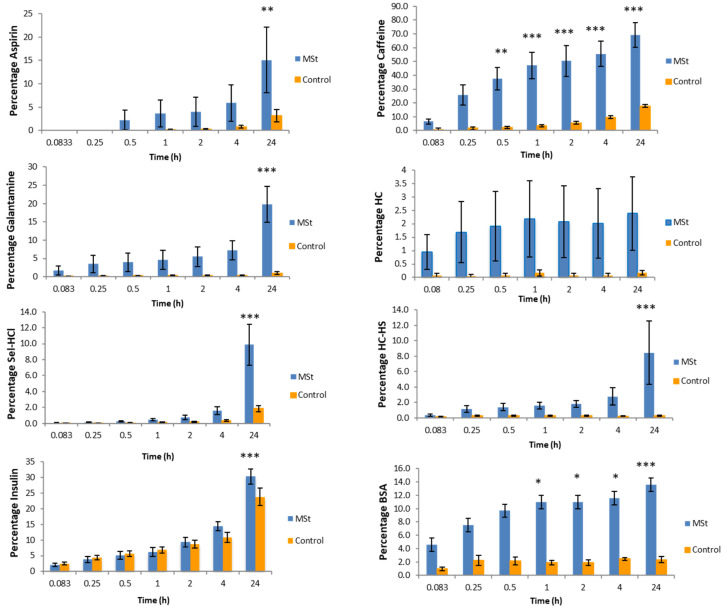
Percentage of total drug present in the receptor chamber over 24 h. Values are ± standard error. Significance difference (*p* ≤ 0.05 one-way ANOVA) is indicated by (*). *p* ≤ 0.01 and *p* ≤ 0.001 are indicated by (**) and (***) respectively (*n* = 9).

Figure 6 shows percentage permeation of each drug diffused from the donor compartment of the Franz Cell into the receiver compartment after 24 h. The greatest percentage permeation was observed with caffeine with 70 ± 8% and the lowest was observed with HC with 2.4 ± 1.3% permeation.

Using the linear portion of each curve, the highest flux obtained was for BSA (8133 ± 1365 μg/cm^2^/h) and the lowest was for HC (11 ± 4 μg/cm^2^/h). BSA and HC also showed the highest (16275 ± 3078 μg) and the lowest (73 ± 47 μg) permeation after 24 h respectively.

MSt enhanced the permeability of all tested molecules across porcine skin model. HC-HS and Insulin showed the highest (40.4 ± 8.2) and lowest (1.3 ± 0.03) ER respectively.

## 4. Discussion

In this study several molecules were selected to span a range of parameters (partition coefficient, molecular size, acid dissociation constant, melting point and solubility), thought to effect permeability of molecules across the skin [61,62]. Considering that in the in vitro excised skin samples, metabolic activity and blood circulation is absent, the immune system is ceased and regeneration has stopped [15], the permeation of molecules is predominantly based on the diffusion of the molecules into the aqueous receiver of the Franz Cell.

The partition coefficient of a compound is related to its hydrophobicity or hydrophilicity, which is the most important factor in deciding which skin penetration pathway the molecule is most likely to follow. It is expected that hydrophilic molecules partition into the hydrated keratin-filled keratinocytes whereas hydrophobic molecules partition into the lipoidal bilayers. Thus, the hydrophilic molecules are more likely to penetrate via the transcellular (intracellular) pathway, whereas the hydrophobic molecules have greater affinity for permeation via the intercellular pathway [63]. There are many studies which suggest that the increase in hydrophobicity of the molecule increases permeation into the stratum corneum [62,64]. This confirms that the SC lipid bilayer is a rate-limiting barrier for permeation of hydrophilic molecules through this skin layer. However, further increases in hydrophobicity of the molecules can then be a rate-limiting barrier for partitioning into the deeper layer of skin (dermis), which is more aqueous in nature [65,66]. The most favourable partition coefficient condition for a drug to permeate across intact skin is in the intermediate range (LogP_(octanol/water)_ of 1 to 3) [61,67,68,69]. Molecules within this range of partition coefficient are hydrophobic enough to absorb into the lipid chain domains of the stratum corneum and the epidermis but also hydrophilic enough to partition into the dermis, which contains a greater percentage of water. In the current study caffeine, with logP −0.07, which is outside the 1–3 logP window recommended, had the highest percentage permeation (70 ± 8%). Thus, molecules with a logP value outside the recommended range are still able to permeate skin effectively with the aid of MSt. In the case of caffeine, it is suggested that the small molecular size has a significant influence on its permeability.

The molecular size (molecular weight) of the drugs is the second most important factor affecting the permeability of drug across the skin. The molecular size has a reverse correlation to the flux of the molecules, where an increase in the size of the molecules reduces the permeability [14,70]. Most established therapeutic drugs for transdermal delivery are small organic molecules, which are more favourable for transdermal delivery and they lie within a narrow range of molecular size of 100–500 Da. With respect to this narrow size range, the influence of the molecular weight on permeation of the molecules is of relatively less importance than the influence of the partition coefficient. However, in terms of dealing with much larger molecules such as proteins and peptides, then the effect of molecular size on transdermal permeation is clearly of critical importance, as these large molecules are unable to penetrate through intact skin to a great extent. In the current study, BSA with a molecular weight of 66 kDa showed the second highest percentage permeation (14 ± 2.5) suggesting that molecules outside the 100–500 Da range are able to permeate effectively with the aid of MSts. The high aqueous solubility of BSA (300 mg/mL) facilitates a small percentage of molecules to permeate without the aid of MSts in vitro. However, this amount is significantly less than when MSts are used.

Ionised drugs are likely to be transported through the skin via the shunt route, whereas a non-ionised drug would be expected to pass predominantly via the lipid intercellular pathway [15]. This suggests that ionised molecules have a tendency to attract to the polar head groups of the lipid domains in the *stratum corneum* and are poor transdermal permeants [71,72]. There are debates against using weak bases and weak acids for transdermal drug delivery, since they can dissociate to varying degrees, depending on the pH of the formulation [15]. The pH of the skin also plays a significant role in relation to the acidity or basicity of drugs. Human skin pH, normally mildly acidic at pH 5.5, creates charged molecules, which consequently affect their permeation [15]. This acidity of skin is related to sebum released from the sebaceous gland of live skin, which in case of excised skin is less relevant. In the current study we used a weak acid drug (aspirin) with pKa 3.5, which is the most dissociated drug tested in this study. Despite aspirin being a weak acid, it is known as one of the most acidic drugs (with low pKa) with quite severe side effects on the gastrointestinal tract, which makes transdermal aspirin delivery highly attractive. Although this study showed aspirin took longer (30 min) to permeate than other tested molecules (5 min), it still showed 15 ± 7% permeation with the aid of MSts compared with 3 ± 1% permeation without the MSts. The slower initial rate of permeation could be due to low aqueous solubility of aspirin (7.5 mg/mL) and negligible diffusion of this drug before 30 min, is likely to be related to the difficulty in detecting aspirin concentrations below a threshold detection limit.

There is a reverse correlation between the melting point and aqueous solubility of molecules. Molecules with high melting point tend to have low solubility in water at normal pressure and temperature (transdermal delivery conditions) [15,73]. In the current study, the drugs used have appropriate melting point to be used in the formulations for transdermal delivery. No drugs with a very high melting point are produced and the effect of this parameter therefore could not be accounted for.

Drugs with poor aqueous solubility do not fully dissolve in the aqueous formulation and thus, the amount of the topically applied drug presented on the skin surface will be relatively small. As the drug absorbs into the skin layers, the concentration gradient created by drug diffusion into the intact *stratum corneum* would oppose further diffusion from the topical application. Thus, drugs which remain trapped in the lipophilic *stratum corneum* can inhibit further drug diffusion [15]. The current study showed that aqueous solubility of drugs is the most important factor in in vitro transdermal drug delivery in combination with MSts. HC, which is insoluble in water, showed lowest permeation percentage (2.3 ± 1.3%) with the aid of MSts. In comparison, the HC-HS with aqueous solubility of 100 mg/mL showed approximately four times higher percentage permeation (8.4 ± 4%), which directly illustrates the effect of solubility on permeation. At the opposite end of the solubility scale BSA with solubility of 300 mg/mL showed intermediate permeation (14 ± 2.5%) despite its very large size.

Several publications have reported transdermal Insulin delivery in vivo. In this study MSts showed no significant enhancement in permeation of Insulin in vitro. Insulin is completely insoluble in water with neutral pH and requires an acidic environment to dissolve (0.01 M HCl, recommended solvent for Insulin by the supplier Sigma Aldrich). It is suggested that using an acidified solution for both dissolving the drug and in the receiver compartment of the Franz cell disrupted the integrity of the skin membrane. Thus, this allows similar levels of permeation of insulin with (30 ± 2.4%) and without (23 ± 2.8%) MSts.

## 5. Conclusions

MSt treatment significantly enhanced the permeation of all the selected molecules with different physicochemical characteristics, in comparison to transport through intact skin. MSts were used to enhance the quantity of delivered molecule by up to 40 times that of topical application with no MSts. MSt-treated skin exhibits greatly increased permeation. The molecule parameters (size, acid dissociation constant, partition coefficient and solubility)—traditional hurdles associated with passive diffusion through intact skin—are overcome using MSt skin treatment. For instance, using MSts proteins and large hydrophilic biologics can be effectively be delivered into skin—which is not possible using conventional topical formulations or patches. MSts render the size of molecules to be of relatively low importance with regards to transdermal transport. Rather, aqueous solubility plays a much more critical role in in vitro skin permeability via MSt microchannels, due to diffusion related factors in the more aqueous based viable epidermis and dermis. Aqueous solubility, together with related parameters such as acid dissociation constant and partition coefficient, played a dominant role in in vitro delivery using MSts. MSt treatment, in combination with topical drug application is thus suggested to enable more efficacious transdermal delivery of a much wider range of drug molecules than has previously been possible using topical applications to intact skin. It is suggested that further optimisation of the MSt platform would increase transdermal drug delivery performance.

## Figures and Tables

**Table 1:** Physicochemical characteristics of mosaic molecules.

Drug Name	Molecular Size (Da)	Partition Coefficient (LogP)	Acid Dissociation Constant (pKa)	Melting Point	Solubility in Water pH 7.4 (mg/mL)
Aspirin	180	1.2	3.5	135	7.5
Galantamine	368	1.8	8.2	126	20
Sel-HCl	223	2.8	6.88	142	100
Insulin	5808	-	-	80	Insoluble
Caffeine	194	−0.07	14	235	20
HC	362	1.6	13.81	220	Insoluble
HC-HS	484	1.6	5.64	-	100
BSA	66,000	-	-	56	300

**Table 2:** Franz cell experimental condition for mosaic molecules.

Drug	Solvent	Solubility (mg/mL)	Franz Cell Receptor Fluid	Initial Drug Quantity on Franz Cell (μg)
Aspirin	PBS	7.5	PBS	3000
Galantamine	PBS	20	PBS	6000
Sel-HCl	PBS	100	PBS	20,000
Insulin	0.01 M HCL	12.5	0.01 M HCL	5000
Caffeine	PBS	20	PBS	2000
HC	Ethanol	12.5	PBS	3750
HC-HS	PBS	100	PBS	20,000
BSA	PBS	300	PBS	120,000

**Table 3:** HPLC conditions for each molecule.

Drug	Mobile Phase	Run Time (min)	Retention Time (min)	Wavelength (nm)
Aspirin	(A) methanol 30%, (B) 0.1% acetic acid 70%	20	8.5	210
Galantamine	(A) acetonitrile 25%, (B) 0.1% TFA 75%	10	2.4	220
Sel-HCl	(A) Methanol 35%, (B) 0.1% acetic acid 65%	10	2.2	210
Insulin	(A) Acetonitrile 70%, (B) 0.1% TFA 30%	15	2.2	210
Caffeine	(A) methanol 55%, (B) 0.1% acetic acid 45%	5	2.4	274
HC	(A) methanol 70%, (B) 0.1% acetic acid 30%	6	2.5	254
HC-HS	(A) Methanol 70%, (B) 0.1% acetic acid 30%	10	4.5	254

**Table 4:** Cumulative amount of molecules, permeated after 24 h through porcine skin (1.77 cm^2^), flux, permeation percentage and enhancement rate (ER).

Drug Name	Cumulative Amount at 24 h (μg)		Flux (μg/cm^2^/h)		Permeation at 24 h (%)		ER
	+ MSt	Control	+ MSt	Control	+ MSt	Control	
Aspirin	452 ± 210	134 ± 38	77 ± 35	3.4 ± 0.5	15 ± 7	3 ± 1	4.8 ± 0.8
Galantamine	1188 ± 290	67 ± 23	49 ± 8	0.7 ± 0.6	20 ± 5	1 ± 0.4	21 ± 3.6
Sel-HCl	1974 ± 511	373 ± 77	43 ± 12	11 ± 2	10 ± 2.5	1.9 ± 0.4	5.2 ± 0.2
Insulin	1515 ± 120	1188 ± 137	85 ± 8	89 ± 6	30 ± 2.4	23 ± 2.8	1.3 ± 0.03
Caffeine	1383 ± 176	356 ± 149	308 ± 18	26 ± 5	70 ± 8	18 ± 7	4.2 ± 1.3
HC	73 ± 47	6 ± 3	11 ± 4	3 ± 0.7	2.3 ± 1.3	0.1 ± 0.08	22.3 ± 10.8
HC-HS	1691 ± 818	53 ± 13	64 ± 21	2 ± 0.03	8.4 ± 4	0.2 ± 0.06	40.4 ± 8.2
BSA	16275 ± 3078	2792 ± 576	8133 ± 1365	188 ± 19	14 ± 2.5	2 ± 0.5	7.1 ± 0.5
